# Co-Cropping Indian Mustard and Silage Maize for Phytoremediation of a Cadmium-Contaminated Acid Paddy Soil Amended with Peat

**DOI:** 10.3390/toxics9050091

**Published:** 2021-04-21

**Authors:** Sifan Wang, Yong Liu, Khalil Kariman, Jialin Li, Huihua Zhang, Fangbai Li, Yinglong Chen, Chongjian Ma, Chuanping Liu, Yuzhen Yuan, Zhiqiang Zhu, Zed Rengel

**Affiliations:** 1 Guangdong General Station of Agricultural Environment Protection and Rural Energy Resource, Guangzhou 510500, China; sifanwangsifan@hotmail.com; 2 National-Regional Joint Engineering Research Center for Soil Pollution Control and Remediation in South China, Guangdong Key Laboratory of Integrated Agro-Environmental Pollution Control and Management, Institute of Eco-Environmental and Soil Sciences, Guangdong Academy of Sciences, Guangzhou 510650, China; jialinlijialin@hotmail.com (J.L.); hhzhang@soil.gd.cn (H.Z.); cefbli@soil.gd.cn (F.L.); cpliu@soil.gd.cn (C.L.); msyzyuan@soil.gd.cn (Y.Y.); 3 The Institute of Agriculture, UWA School of Agriculture and Environment, The University of Western Australia, Perth, WA 6009, Australia; khalil.kariman@uwa.edu.au (K.K.); yinglong.chen@uwa.edu.au (Y.C.); zed.rengel@uwa.edu.au (Z.R.); 4 Henry Fok College of Biology and Agriculture, Shaoguan University, Shaoguan 512005, China; ma_chj@hotmail.com; 5 College of Tropical Crops, Hainan University, Haikou 570228, China; 6 Institute for Adriatic Crops and Karst Reclamation, 21000 Split, Croatia

**Keywords:** co-cropping, cadmium, Indian mustard, silage maize, plant density, peat application, phytoremediation

## Abstract

Co-cropping is an eco-friendly strategy to improve the phytoremediation capacity of plants growing in soils contaminated with heavy metals such as cadmium (Cd). This study was conducted to investigate the effects of co-cropping Indian mustard (*Brassica* *juncea*) and silage maize (*Zea* *mays*) and applying peat on the phytoremediation of a Cd-contaminated acid paddy soil via characterizing plant growth and Cd uptake in pot experiments. There were six planting patterns (Control: no plants; MI-2 and MI-4: mono-cropping of Indian mustard at low and high densities, respectively; MS: mono-cropping of silage maize; CIS-2 and CIS-4: co-cropping of Indian mustard at low and high densities with silage maize, respectively) and two application rates of peat (NP: 0; WP: 30 g kg^−1^). When Indian mustard and silage maize were co-cropped, the shoot biomass of Indian mustard plants per pot was significantly (*p* < 0.05) lower than that obtained in the mono-cropping systems, with a substantial reduction (55–72%) in the same plant density group. The shoot biomass of silage maize plants in the mono-cropping systems did not differ significantly from that in the co-cropping systems regardless of the density of Indian mustard. The growth-promoting effect of the peat application was more pronounced in Indian mustard than silage maize. Under the low density of Indian mustard, the co-cropping systems significantly (*p* < 0.05) decreased Cd uptake by silage maize. Additionally, soil amendment with peat significantly (*p* < 0.05) increased shoot Cd removal rate and Cd translocation factor value in the co-cropping systems. Taken together, the results demonstrated that silage maize should be co-cropped with Indian mustard at an appropriate density in Cd-polluted soils to achieve simultaneous remediation of Cd-contaminated soils (via Indian mustard) and production of crops (here, silage maize). Peat application was shown to promote the removal of Cd from soil and translocation of Cd into shoots and could contribute to enhanced phytoremediation of Cd-contaminated acid paddy soil.

## 1. Introduction

Cadmium (Cd) is a non-essential element. Because of its high mobility, Cd easily enters into the food chain and adversely affects human health due to its high biotoxicity [1,2]. According to Wang et al. [3], during 2001–2010, anthropogenic activities such as smelting, mining, waste disposal, pesticide and fertilizer application, and emissions from motor vehicles led to a substantial increase in Cd contamination of soils in China. Additionally, China’s 2014 report on the “National General Survey of Soil Contamination” delivered by the Ministry of Environmental Protection and the Ministry of Land and Resources revealed that the total soil pollution rate in China is 16% higher than the Chinese Environmental Quality Standard for Soils (GB 15618-1995), with Cd accounting for almost half (7%) of that increase, ranking the first among inorganic pollutants [4]. Recent Cd-related food safety incidents (“Cd rice” and “Cd wheat”) has generated widespread awareness of Cd contamination of farmland soils and the safety of agricultural products [5]. Therefore, the remediation of Cd-contaminated farmland soils is a high-priority environmental challenge that must be urgently addressed.

Phytoremediation is an eco-friendly and cost-effective strategy that uses metal-hyperaccumulating plants to extract heavy metals from contaminated soils [5,6]. However, using hyperaccumulators alone to remediate Cd-contaminated farmland soils can be uneconomic and inefficient. Generally, the efficiency of phytoremediation of Cd-contaminated farmland soils is strengthened by the use of appropriate agronomic and chemical measures. Furthermore, simultaneous remediation of Cd-contaminated farmland soils and production of crops can be advantageous environmentally and economically. To achieve this goal, co-cropping (e.g., inter-cropping and mixed cropping) techniques using hyperaccumulators along with some crops are often applied. For example, Luo et al. [7] showed that co-cropping the hyperaccumulator *Sedum plumbizincicola* and the main crop, celery (*Apium graveolens*), increased Cd uptake by *S*. *plumbizincicola* while reducing Cd accumulation by celery.

Some metal-accumulating crop plants have the advantages of large biomass, well-developed main roots, and strong adaptability [8]. Co-cropping of such accumulators with other crops has attracted increasing attention. For instance, higher Cd uptake and phytoremediation efficiency were achieved in a co-cropping system using Indian mustard (*Brassica juncea*) and oilseed rape (*Brassica napus*) compared with mono-cropped Indian mustard or oilseed rape [9]. Co-cropping of amaranth (*Amaranthus paniculatus*) with maize (*Zea mays*) promoted Cd uptake by amaranth while decreasing Cd accumulation by maize [10]. Among the accumulators mentioned above, Indian mustard has the fast growth and relatively large biomass production [8]. On the other hand, maize is an important main crop in South China, which can be cultivated for silage three times a year [11]. Hence, there is a potential of co-cropping Indian mustard and silage maize for phytoremediation of Cd-contaminated soils; however, the available knowledge on this topic is quite limited.

To enhance Cd phytoextraction, chelate-induced uptake of Cd from contaminated farmland soils has been studied extensively. For example, the addition of the chelating agent ethylenediaminetetraacetic acid (EDTA) enhanced the translocation of Cd from the roots of Indian mustard to its shoots; however, the addition of EDTA did not alter the total Cd uptake by plants but increased the environmental risk of Cd leaching [12]. Wu et al. [11] added a mixture of chelators (monosodium glutamate waste liquid, citric acid and EDTA) to a Cd-contaminated paddy soil co-cropped with the hyperaccumulator *Sedum alfredii* Hance and the low metal-accumulating maize, which led to the highest phytoextraction rate of Cd. Shen et al. [13] found that the application of a chelator (EDTA or citric acid) combined with peat effectively promoted the translocation of Cd from the roots of ramie (*Boehmeria nivea*) to its shoots and improved the removal efficiency of Cd from soil. However, Eriksson [14] observed that peat showed a strong capacity to adsorb Cd and reduce its bioavailability in soil, and thus decreased Cd uptake by perennial ryegrass (*Lolium perenne*) and oilseed rape. Hence, whether peat can promote or hamper the phytoremediation of Cd-contaminated soils in co-cropping systems remains unclear.

This study aimed to characterize the effects of co-cropping Indian mustard (as an accumulator) and silage maize (as a main crop), and peat application on plant growth and Cd uptake, in order to evaluate the potential of this co-cropping system to phytoremediate a Cd-contaminated acid paddy soil. We sought to better understand whether the efficiency of phytoremediation change in response to the intensity of inter- (i.e., co-cropping of Indian mustard with silage maize) and intra-specific competition (i.e., increasing Indian mustard density). We further focused on the influences of peat application on the removal of Cd from soil and translocation of Cd into shoots in the co-cropping system. Using phytoremediation coupled with peat application, we hypothesized that Indian mustard at an appropriate plant density and co-cropped with silage maize may reduce Cd uptake by silage maize without affecting the main crop productivity and achieve higher phytoremediation efficiency when peat was amended to the Cd-contaminated soil.

## 2. Materials and Methods

### 2.1. Materials

The acid paddy soil (silty clay loam) was collected from a heavily Cd-contaminated rice field (23°31′50″ N, 114°23′08″ E) in Huizhou City, Guangdong province, China. Woody peat (hereinafter referred to as peat) used in this study was purchased from Zhongxiang Xuyao Science and Technology Co., Ltd. (Jiangyin, China). The peat was originally derived from a tropical peat swamp forest in Indonesia. According to Posa et al. [15], the peat developed in that area is a stable, organic material composed of partially decayed woody plant debris and characterized by long-term carbon accumulation under high-temperature and high-rainfall conditions. The collected soil and peat were air-dried and passed through a 2-mm nylon sieve. Basic physicochemical properties (analyzed using the methods detailed in Lu [16]) of the soil and peat are listed in Table 1. The seeds of Indian mustard (*Brassica juncea*), a metal-accumulating crop, were purchased from Wuhan Angu Agricultural Science and Technology Co., Ltd. (Wuhan, China). The seeds of silage maize (*Zea mays* var. Yunshi No. 5), a main crop with low metal-accumulation capacity, were obtained from the Yunnan Academy of Agricultural Sciences (Kunming, China).

### 2.2. Experimental Set-Up

The pot experiments were conducted in a glasshouse (23°10′59″ N, 113°21′01″ E) in the Institute of Eco-environmental and Soil Sciences, Guangdong Academy of Sciences. The sealed pots were made of a PVC tube (20-cm inner diameter and 22-cm height). The experiment comprised two factors (i.e., planting pattern and peat application) at various levels. There were six planting patterns altogether, including no plants (Control), mono-cropping of Indian mustard at a low density (2 plants pot^−1^) (MI-2), mono-cropping of Indian mustard at a high density (4 plants pot^−1^) (MI-4), mono-cropping of silage maize (MS), co-cropping of Indian mustard at a low density (2 plants pot^−1^) with silage maize (CIS-2), and co-cropping of Indian mustard at a high density (4 plants pot^−1^) with silage maize (CIS-4). Peat was applied at rates of 0 (NP) and 30 g kg^−1^ (WP). Thus, there were two non-planted controls (C_1_ and C_2_) and 10 planted treatments (T_1_–T_10_) as detailed in Table 2. The experiment was carried out in triplicate, and pots were re-positioned periodically to minimize any effects of environmental gradients.

All the treatment and control groups received a compound fertilizer (N:P_2_O_5_:K_2_O = 20:5:20, *w*/*w*) and superphosphate (P_2_O_5_ = 12%, *w*/*w*) that are commonly used as basal fertilizers in local farming practices. Each pot was filled with 6 kg of air-dried soil and mixed with the compound fertilizer and superphosphate fertilizer to achieve the following application rates: N, 100 mg kg^−1^; P_2_O_5_, 80 mg kg^−1^; and K_2_O, 100 mg kg^−1^. Subsequently, half of the soil was supplemented with peat at the application rate of 30 g kg^−1^.

After soil homogenization, the pots were incubated in the glasshouse for 18 d, with soil moisture maintained at 60% water-holding capacity (WHC). After soaking in the deionized water for 8 h, eight Indian mustard seeds and/or three silage maize seeds were initially sown per pot, followed by thinning to two or four Indian mustard plants and/or one silage maize plant per pot 15 d after sowing. The two control groups (with and without peat supplementation) were prepared simultaneously without any plants. Pots were watered every 2 days using deionized water to maintain 60% WHC. The plants were harvested at day 90 after sowing and separated into shoots and roots.

After harvest, homogenous soil subsamples were taken from all treatments. The collected soil samples were air-dried, ground and sieved through a 2-mm nylon sieve.

### 2.3. Sample Preparation and Analysis

At harvest, shoots were separated from roots by cutting at the shoot–root junction with stainless-steel scissors; then, the intact and broken sections of the roots were carefully removed from the soil. The collected shoots and roots were thoroughly washed with tap water, rinsed with deionized water, and gently blotted with tissue paper. Samples were placed in an oven at 105 °C for 30 min and then at 75 °C for several days to achieve constant dry weight. Dried shoot and root samples were ground in a stainless-steel grinder and passed through a 1-mm nylon sieve. To compare growth performance and plant-plant interactions, the following indexes were calculated [17,18,19]:

Root/shoot (R/S) ratio = dry root biomass/dry shoot biomass

RCI_inter_ = (Size_mono_ × SP_p_ − Size_co_)/(Size_mono_ × SP_p_) or (Size_mono_ × SP_q_ − Size_co_)/(Size_mono_ × SP_q_)

RCI_intra_ = (Size_ld_ − Size_hd_)/Size_ld_

where RCI_inter_ and RCI_intra_ represent the relative competition intensity (RCI) of inter- and intra-specific interactions, respectively. Size_mono_ and Size_co_ are measures of individual size of a given plant species under the same plant density group in the mono- and co-cropping systems, respectively, and SP_p_ and SP_q_ are sown proportions of Indian mustard (p = 2/3 or 4/5) and silage maize (q = 1/3 or 1/5), respectively, in a co-cropping system (p + q = 1). Size_ld_ and Size_hd_ are individual sizes of Indian mustard in the mono-cropping systems in the low- (ld) and high-density (hd) groups, respectively. If the RCI value is positive, the inter- or intra-specific interaction has a competitive effect on the species; if the RCI value is negative, the inter- or intra-specific interaction has a facilitative effect on the species. Shoot biomass per plant for each species, which was suggested as the best parameter to assess inter- or intra-specific interaction [20], was used as a proxy for individual size in the present study.

The Cd concentration in plant samples was determined by graphite furnace atomic-absorption spectrometry using a PerkinElmer PinAAcle 900Z instrument (Shelton, CT, USA) after HNO_3_–HClO_4_ digestion [16]. Cadmium accumulation (dry biomass × Cd concentration) and removal rate (Cd accumulation in plant material/total amount of soil Cd) were calculated for shoots, roots and whole plants [9,11]. The Cd translocation factor (TF_Cd_) was calculated as follows [21]:

TF_Cd_ = amount of Cd in shoots/total amount of Cd in plants.

The pH values of the soil samples (1:2.5 soil-to-water ratio, *w*/*v*) were measured by potentiometry using a Sartorius PB-10 pH meter equipped with a Sartorius pH/ATC electrode (Göttingen, Germany) [16]. The available Cd (DTPA-extractable) concentration in soil samples was determined by inductively coupled plasma optical emission spectrometry using a PerkinElmer Optima 8000 instrument (Shelton, CT, USA) after extraction with diethylenetriaminepentaacetic acid (DTPA) at a 1:5 soil-to-solution ratio (*w*/*v*) [16].

The certified reference materials (GBW 10020 for plant and GBW 07429 for soil, purchased from the Institute of Geophysical and Geochemical Exploration, Chinese Academy of Geological Sciences, Langfang, China) were also analyzed. The Cd recovery in these two certified reference materials ranged from 98% to 110%.

### 2.4. Data Analysis and Statistics

To determine the significance (*p* value) of main effects and interactions, the two-way analysis of variance (ANOVA) was performed using planting pattern (Pattern) and peat application (Peat) as the main factors. The Shapiro–Wilk test showed that the residuals for all measured parameters were (close to being) normally distributed. Also, no extreme outliers were detected by boxplots. Multiple comparisons of means were carried out using the Tukey test. Pairwise comparisons of means were performed using the independent samples *t*-test. Calculation of Pearson’s correlation coefficient (*r* value) was done using a bivariate correlation analysis followed by the two-tailed *t*-test of significance.

## 3. Results

### 3.1. Soil pH and Available Cd

The ANOVA results for soil pH and available Cd are given in Table 3. Both Pattern and Peat significantly (*p* < 0.01) affected soil pH, but no significant interaction between the two factors was found. The ANOVA results also revealed that the Pattern × Peat interaction exhibited a significant (*p* < 0.05) effect on the DTPA-extractable Cd concentration in soil.

**Figure 1:**
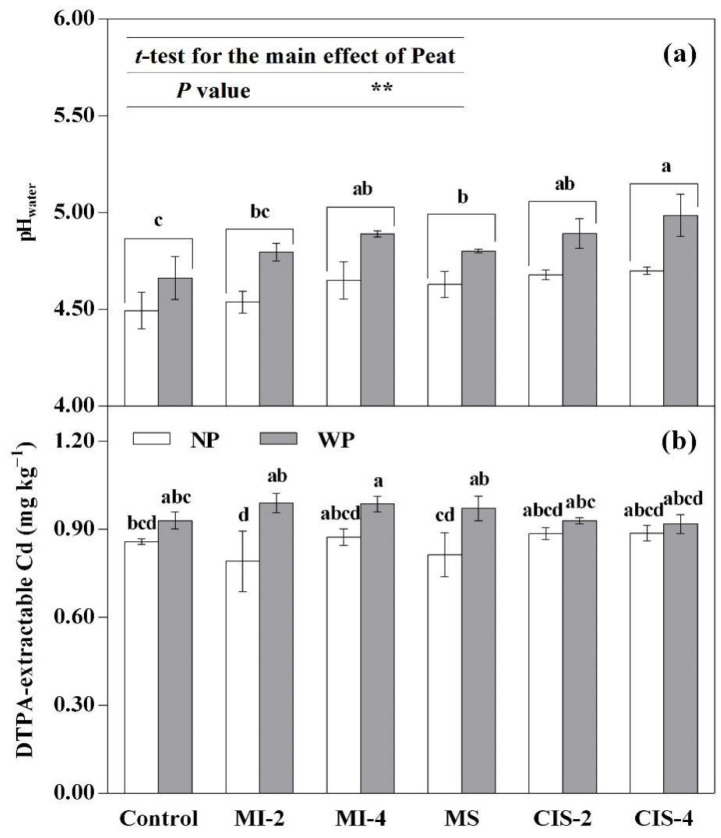
Effects of planting pattern and peat application on pH_water_ (**a**) and DTPA-extractable Cd (**b**) in soil. For explanation of treatment notations, see Table 2; data are expressed as means ± SD (*n* = 3); different lowercase letters (e.g., a, b, c, etc.) indicate significant (*p* < 0.05) differences among the planting patterns (the interaction between planting pattern and peat application was non-significant) or among the treatments based on the Tukey test; the asterisk indicates a highly significant (** *p* < 0.01) difference between the NP and WP treatment groups.

Regarding soil pH, the results of the Tukey test and *t*-test are presented in Figure 1a. Compared with the non-planted control, the soil pH increased significantly (*p* < 0.05) in almost all planting patterns (except MI-2). However, in a given MI or CIS system, the soil pH did not significantly change in the high-density in comparison with low-density groups. In addition, in a given plant density group, there was no significant difference in soil pH between the MI and CIS systems. The peat application increased soil pH significantly (*p* < 0.01).

The soil DTPA-extractable Cd concentration increased significantly (*p* < 0.05) with the application of peat in the MI-2 and MS treatments (Figure 1b).

### 3.2. Shoot and Root Biomass of Indian Mustard and/or Silage Maize

The interaction had a significant (*p* < 0.05) effect on the shoot biomass of Indian mustard, whereas only the main effect of Peat influenced silage maize shoot biomass (*p* < 0.01) (Table 4). Although there was no significant interaction between Pattern and Peat, the effect of both main factors on the total root biomass of plants (Indian mustard and/or silage maize) was significant (*p* < 0.01 or <0.05) (Table 4). Both Pattern and Peat influenced the R/S ratio significantly (*p* < 0.01 or <0.05), but the interaction was not significant (Table 4).

**Figure 2:**
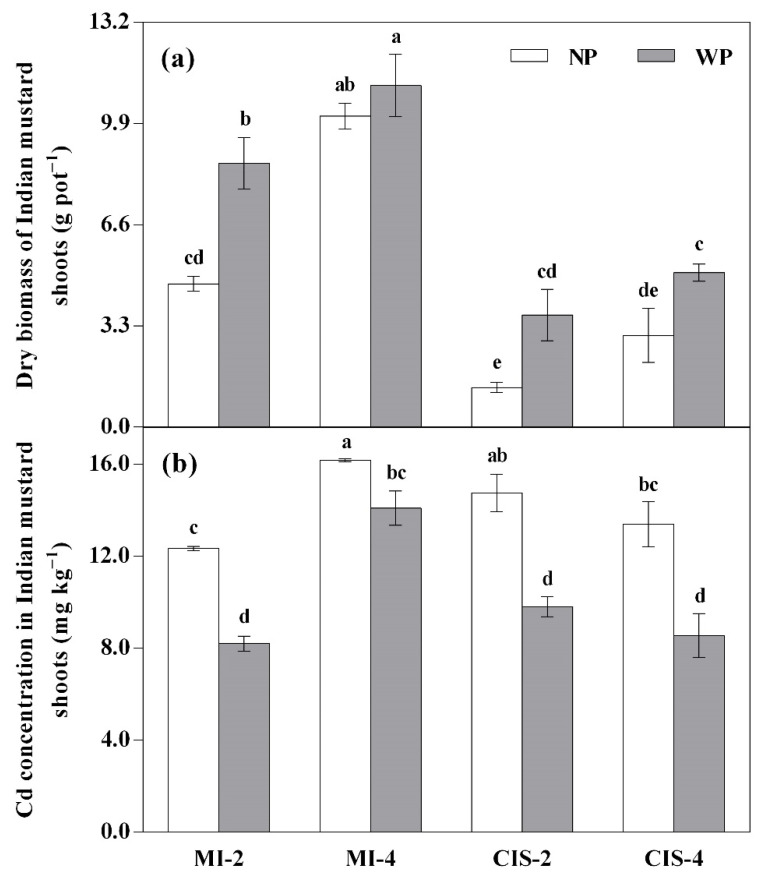
Effects of planting pattern and peat application on dry biomass (**a**) and Cd concentration (**b**) in Indian mustard shoots. For explanation of treatment notations, see Table 2; data are expressed as means ± SD (*n* = 3); different lowercase letters (e.g., a, b, c, etc.) indicate significant (*p* < 0.05) differences among the treatments based on the Tukey test.

Regarding Indian mustard shoot biomass (Figure 2a), with or without peat amendment, the MI-4 treatment always yielded greater shoot biomass than the other treatments, showing significant (*p* < 0.05) increases of 116–683% for the NP and 29–203% for the WP treatments. In a given plant density group, the CIS treatments generally produced smaller shoot biomass than the MI treatments, showing significant (*p* < 0.05) decreases of 70–72% in the NP and 55–57% in the WP treatments.

Table 5 shows silage maize shoot biomass in different treatments. No significant difference was found in the shoot biomass of silage maize between the MS and CIS systems, regardless of the density of Indian mustard. The application of peat significantly (*p* < 0.01) promoted silage maize shoot growth (average increase of 17%).

The total root biomass of plants was ranked in the order CIS-4 > CIS-2 and MS > MI-4 > MI-2 (Supplemental Table S1). Accordingly, in a given MI or CIS system, the high-density groups had significantly (*p* < 0.05) greater total root biomass than the low-density groups; in a given plant density group, the CIS systems had significantly (*p* < 0.05) greater total root biomass than the MI systems. The Peat’s main effect did not significantly influence the total root biomass.

With or without peat amendment, the MS and CIS systems had significantly (*p* < 0.05) higher R/S ratios than the MI systems (Supplemental Table S2). Increased plant density did not significantly affect the R/S ratio, regardless of Pattern and Peat. Moreover, the R/S ratio of plants did not change significantly regardless of the peat application.

### 3.3. Cd Uptake in Shoots and Roots of Indian Mustard and/or Silage Maize

The ANOVA results for Cd concentration and accumulation in shoots and roots of Indian mustard and/or silage maize are presented in Table 4. The interaction had a significant (*p* < 0.01) effect on Cd concentration in shoots of Indian mustard, whereas Cd accumulation in shoots of Indian mustard plants was significantly (*p* < 0.01) influenced only by Pattern. Similar observations were recorded on the concentration and accumulation of Cd in the total roots. The main effects of both Pattern and Peat on the concentration and accumulation of Cd in silage maize shoots were significant (*p* < 0.01), but the interaction was not.

Compared with the MI-2 treatments, Cd concentration in shoots of Indian mustard in the CIS-2 treatments was significantly (*p* < 0.05) increased in the NP but not in the WP treatments (Figure 2b). However, with or without peat amendment, the MI-4 treatment had significantly (*p* < 0.05) higher shoot Cd concentration than the CIS-4 treatment in both NP and WP treatments. Furthermore, in the MI treatments, increased plant density led to significant (*p* < 0.05) increases of 32% in the NP and 72% in the WP treatments in shoot Cd concentrations, whereas in the CIS treatments increased plant density did not significantly affect shoot Cd concentration regardless of the peat application.

Cadmium accumulation in Indian mustard shoots followed the order MI-4 > MI-2 > CIS-4 > CIS-2 (Supplemental Figure S1). Hence, in a given MI or CIS system, the shoot Cd accumulation was significantly (*p* < 0.05) higher in the high-density than low-density groups; in a given plant density group, the shoot Cd accumulation was significantly (*p* < 0.05) higher in the MI than CIS systems.

The highest silage maize shoot Cd concentration was in MS, followed by CIS-4 and CIS-2 (*p* < 0.05) (Table 5). The same trend was observed in silage maize shoot Cd accumulation (Table 5). Shoot Cd concentration did not change significantly regardless of the peat application; however, silage maize shoots accumulated significantly (*p* < 0.05) more Cd in the WP relative to NP groups (average increase of 35%).

**Figure 3:**
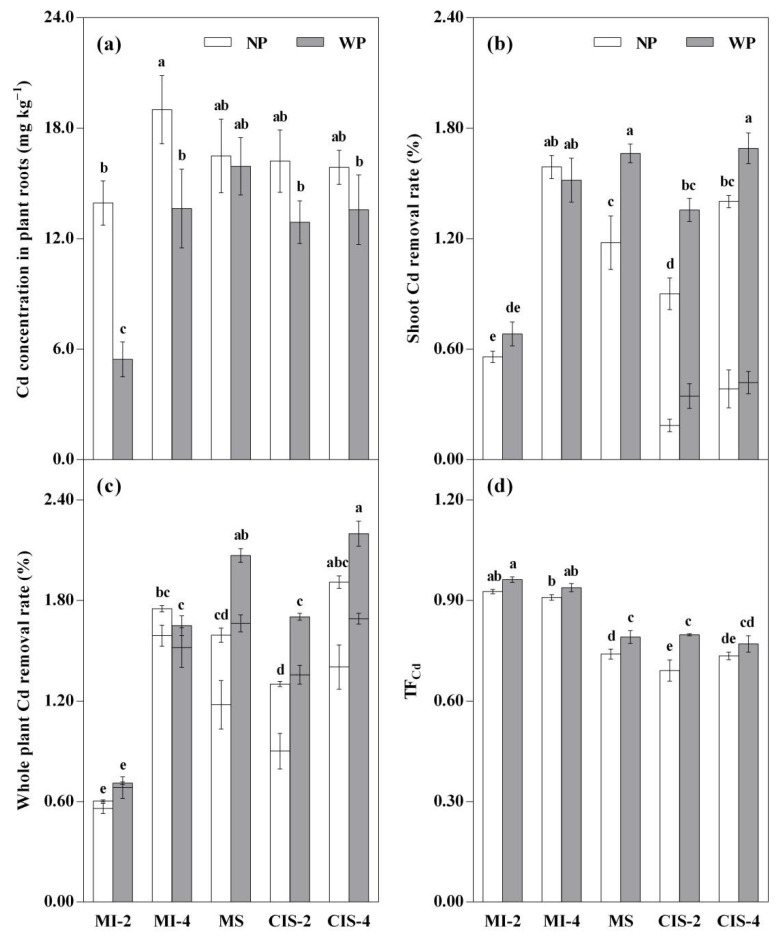
Effects of planting pattern and peat application on root Cd concentration (**a**), shoot Cd removal rate (**b**), whole plant Cd removal rate (**c**), and Cd translocation factor (TF_Cd_) (**d**) of Indian mustard and/or silage maize plants. For explanation of treatment notations, see Table 2; data are expressed as means ± SD (*n* = 3); the lower and upper parts of the stacked columns represent the shoot Cd removal rates of Indian mustard and silage maize plants, respectively (**b**) and the Cd removal rates by shoots and roots, respectively (**c**); different lowercase letters (e.g., a, b, c, etc.) indicate significant (*p* < 0.05) differences among the treatments based on the Tukey test.

The MI_2_WP treatment had significantly (*p* < 0.05) lower root Cd concentration than the other treatments (Figure 3a). With or without peat amendment, in the MI treatments, the root Cd concentration significantly (*p* < 0.05) increased with increasing density of Indian mustard, whereas in the CIS treatments increased plant density did not significantly influence root Cd concentration. In addition, in the MI-2 and MI-4 treatments, root Cd concentration significantly (*p* < 0.05) decreased with the application of peat.

The MI systems had significantly (*p* < 0.05) lower root Cd accumulation compared with the MS and CIS systems (Table S1). Moreover, root Cd accumulation in the MI or CIS systems increased significantly (*p* < 0.05) with the increasing density of Indian mustard.

### 3.4. Removal of Cd from Soil by Indian Mustard and/or Silage Maize and Translocation to Shoots

In the present study, the removal rate of Cd was considered to be a comprehensive index of phytoremediation efficiency of Cd-contaminated soil, which was based on the Cd accumulation in shoots, roots or whole plants as a percentage of the total amount Cd in soil. The translocation of Cd from roots to shoots was indicated by TF_Cd_.

The interaction had a significant (*p* < 0.01) effect on the Cd removal rate in shoots or whole plants, as well as on the TF_Cd_, but the root Cd removal rate was influenced significantly (*p* < 0.01) only by Pattern (Table 4).

The removal rates of Cd in shoots (Figure 3b) and whole plants (Figure 3c) revealed the following points: (1) With or without peat amendment, in a given MI or CIS system, the high-density treatments had significantly (*p* < 0.05) higher Cd removal rates than the low-density treatments, whereas in a given low-density group, the Cd removal rates were significantly (*p* < 0.05) higher in the CIS than MI treatments. (2) In the MS and CIS systems, almost all WP treatments had significantly (*p* < 0.05) higher Cd removal rates in comparison to the respective NP treatments (except in CI_4_S_1_WP vs. CI_4_S_1_NP, with no significant difference regardless of the peat application). Additionally, in the CIS systems, the shoot Cd removal rate was lower in Indian mustard than silage maize, with the former accounting for 21–27% of the shoot Cd removal (stacked columns in Figure 3b).

The removal rate of Cd in roots followed the same trend as those obtained in total root biomass and root Cd accumulation, ranking in the order CIS-4 > CIS-2 and MS > MI-4 > MI-2 (*p* < 0.05) (Table S2). In different treatments, the root Cd removal rate of Indian mustard and/or silage maize was lower than that for shoots, with the root Cd removal accounting for 4–31% of the whole plant Cd removal (stacked columns in Figure 3c).

With or without peat amendment, the MI treatments had significantly (*p* < 0.05) higher TF_Cd_ values than the MS and CIS treatments (Figure 3d). Increased plant density did not significantly affect the TF_Cd_, regardless of Pattern and Peat. In the MS and CIS-2 treatments, the TF_Cd_ significantly (*p* < 0.05) increased with the application of peat.

## 4. Discussion

### 4.1. Effects of Peat Application on Soil pH and Available Cd

Organic materials may change soil pH via the following possible mechanisms [22,23]: (1) Decarboxylation of the organic anions increases soil pH. (2) Decomposition of organic materials converts base cation salts into carbonates, producing the alkaline reaction. (3) Ammonification of organic N consumes protons and increases soil pH, whereas the subsequent nitrification processes releases protons, thereby lowering the soil pH. (4) Direct interactions between the organic compounds and soil surfaces could alter the soil pH in dependence on the initial soil pH. In the present study, peat (pH 5.21) amendment significantly (*p* < 0.01) increased soil pH (Figure 1a). Furthermore, both CEC and OM content of the peat (83.0 cmol·kg^−1^ and 462 g·kg^−1^, respectively) in our study were considerably higher than those of the soil (9.82 cmol·kg^−1^ and 24.4 g·kg^−1^, respectively; Table 1). Xu et al. [24] reported positive linear relationships between CEC and OM vs. pH buffering capacity (pHBC). Therefore, peat amendment could increase soil pHBC via increasing the soil CEC and OM content. Accordingly, the possibly higher pHBC in the peat-amended treatments was expected to negate (at least partly) the fertilization-induced acidification of the soil, resulting in increased soil pH.

In addition to the above-mentioned mechanisms involved in the peat-mediated pH effect, the plant-induced pH changes could also result in the net consumption and/or release of protons. Compared with the pH values of the non-planted controls (4.49 for CtrlNP and 4.66 for CtrlWP), the soil pH values in all planted treatments increased by 0.14–0.33 with the application of peat and 0.05–0.21 in the non-peat treatments (Figure 1a).

In the present study, when low-density Indian mustard or silage maize was mono-cropped, soil amendment with peat resulted in a significant (*p* < 0.05) increase in the concentration of DTPA-extractable Cd (Figure 1b). This might have been due to the combined effects of pH, CEC, OM, etc. Presence of peat may affect the available Cd concentration in soil by altering soil pH, cation exchange capacity (CEC), organic matter (OM) content and composition, and other soil physicochemical properties (e.g., soil texture and clay content) [13,14,25,26]. Although some studies have reported a negative relationship between pH and DTPA-extractable Cd in peat-amended soils [25,26], our results showed the application of peat significantly increased soil pH, but the DTPA-extractable Cd concentrations did not decrease as a consequence because pH (even with an increase) remained below 5.0, ensuring relatively high Cd availability (Figure 1). When both peat-amended and non-amended groups were evaluated together, a significant positive correlation was found between pH and DTPA-extractable Cd (*r* = 0.719 **, *n* = 12), which is consistent with the finding of Jafarnejadi et al. [27].

Generally, higher soil CEC implies a greater Cd^2+^ retention capacity [28]. However, when amended with different rates of peat, He and Singh [25] found a positive relationship between soil CEC and DTPA-extractable Cd. In addition, increased DTPA-extractable Cd concentration was not accompanied by increased content of soil OM [26]. Eriksson [14] observed that the application of peat might result in adsorption of Cd in a non-exchangeable form (e.g., formation of strong complexes between Cd^2+^ and organic substances). Hence, the relationship between peat application and Cd availability is complex.

Decomposition of OM may influence the content and composition of dissolved organic matter (DOM) and its complexing effect on Cd in soils, thereby influencing the solubility and environmental behavior of Cd [29]. Peat decomposition may produce various low-molecular-weight fractions of DOM, such as organic acids, sugars and amino acids [30], as the major organic ligands for complexing Cd and thus increasing the solubility and mobility of Cd in peat-amended soils [13,26]. This might have been one of the mechanisms behind the enhanced Cd availability (increased DTPA-extractable Cd) in the peat-amended treatments in the present study (Figure 1b).

### 4.2. Effects of Mono- or Co-Cropping on Plant Growth and Cd Uptake

Competition and facilitation are the two main types of species interactions in inter-cropping systems [31]. Previous studies have shown that when amaranth (*Amaranthus paniculatus*) or mustard oilseed rape (*Brassica juncea*) plants were inter-cropped with maize (*Zea mays*), the growth of maize was inhibited by amaranth but promoted by mustard oilseed rape [10,32]. In the present study, co-cropping Indian mustard and silage maize inhibited the growth of Indian mustard, likely due to lower competition capacity of Indian mustard for environmental resources (e.g., water, nutrients and light).

The root/shoot (R/S) ratio can influence the uptake of water and nutrients (e.g., nitrogen and phosphorus) by plant roots [17]. The R/S ratio can also vary in response to the availability of nutrients in soil [33]. The higher the R/S ratio, the relatively larger fraction of dry matter is allocated to the roots, leading to further development of the root system. In the present study, the R/S ratio of silage maize plants was significantly (*p* < 0.05) higher in the MS systems than that of Indian mustard plants in the MI systems (Table S2). Under such circumstances, it is likely that owing to its relatively well-developed root system (higher R/S ratio), the silage maize plants may exhibit a greater capacity to absorb water and nutrients from the rhizosphere soil than the Indian mustard plants.

The relative inter-species competition intensity (RCI_inter_) can be used to compare the competitive capacity of different plant species, measuring competitive changes within a given species combination [18]. The larger the value of the RCI_inter_, the higher inter-specific competition occurs, leading to a greater decrease in the size of one species. In the present study, the RCI_inter_ value of Indian mustard was greater than that of silage maize in both the low- and high-density groups (Supplemental Figure S3a). As a result, the inter-specific competition exerted a negative effect on the growth of Indian mustard, whereas the growth of silage maize was unaffected.

Inter-cropping with a main crop may affect the Cd uptake of co-cropped plants in all three possible scenarios [10,34]. In this study, for the respective peat-amended and non-amended groups, co-cropped Indian mustard at varying densities reduced the Cd concentration in silage maize shoots compared with the MS systems (Table 5). This was possibly due to inter-specific competition for the absorption of Cd^2+^ by the two plant species. In relation to the rhizosphere effect induced by plant root excretions, the mobilized Cd may be preferentially absorbed by (hyper)accumulators [35]. As a result, the TF_Cd_ value of Indian mustard was significantly (*p* < 0.05) higher than that of silage maize (Figure 3d). However, the root exudates-mediated inter-specific interaction may result in decreased or increased availability of Cd in soil [34,36]. In the present study, co-cropped silage maize differentially influenced the Cd concentration in Indian mustard shoots in a density-dependent manner compared with the MI systems (Figure 2b). In the high-density groups, the significantly (*p* < 0.05) higher RCI_inter_ value (positive) of Indian mustard than RCI_inter_ value (negative) of silage maize (Supplemental Figure S3a) tended to lower Cd uptake by an individual Indian mustard plant, thereby decreasing its shoot Cd concentration. The inverse might have applied in the low-density groups.

The accumulation of Cd in plants is related not only to the Cd uptake capacity, but also depends on their biomass [9,37]. According to our results, when Indian mustard plants were co-cropped with silage maize, the reductions of 55–72% in the shoot biomass of Indian mustard plants resulted in decreases of 50–76% in their shoot Cd accumulations (Supplemental Figure S1). Differently, co-cropping led to decreases of 14–40% in silage maize shoot Cd accumulations without affecting their shoot biomass (Table 5). This would suggest lower Cd uptake by silage maize due to its competition with Indian mustard.

### 4.3. Effects of Indian Mustard Density on Plant Growth and Cd Uptake

Plant density is a key factor determining the magnitude of plant growth and dry matter accumulation. Within a certain plant density range, the total dry matter production increases with increasing leaf area index; however, at higher plant densities, the growth of individual plants can be reduced considerably [19,38]. It is increasingly recognized that the balance between competitive and facilitative interactions can play an important role in determining the relationships between density and biomass in plant populations [39,40].

In the present study, although the shoot biomass of Indian mustard plants per pot increased with increasing plant density (Figure 2a), the density-dependent interaction demonstrated variable responses of Indian mustard shoot biomass per plant in different treatments (Supplemental Figure S2). For the peat-amended treatments, when the plant density increased from 2 to 4 plants per pot, the shoot biomass of individual Indian mustard plants significantly (*p* < 0.05) decreased only in the MI systems (Supplemental Figure S2). Due to its positive relative intra-species competition intensity (RCI_intra_) value in the peat-amended treatment (Supplemental Figure S3b), mono-cropped Indian mustard likely suffered from intra-specific competition more in the high-density than low-density groups. Therefore, the growth-promoting effect of peat application on shoot biomass of individual Indian mustard plants decreased with increasing plant density, especially in the MI_4_WP treatment. In contrast to the peat-amended treatments, the results of non-amended treatments showed that even though the plant density reached 4 plants per pot, the shoot biomass of individual Indian mustard plants did not change significantly in the MI and CIS systems (Supplemental Figure S2). With or without peat amendment, our results obtained in the CIS systems are consistent with the findings of Lin et al. [41] and Ma et al. [42], who observed a similar phenomenon in their inter-cropping studies on the relationship between hyperaccumulator Gallant soldier (*Galinsoga parviflora*) density (2 and 4 plants pot^−1^) and individual shoot biomass. However, the authors also found that, when Gallant soldier plants were inter-cropped with radish (*Raphanus sativus*) or soybean (*Glycine max*), the shoot biomass of individual Gallant soldier plants increased with increasing plant density up to 3 plants per pot [41,42].

Plant density could also affect Cd uptake by plants [41,42]. In the present study, the concentration and accumulation of Cd in Indian mustard shoots generally showed significantly increasing trends with increasing plant density both in the MI and CIS systems (except in CIS-2 vs. CIS-4, the shoot Cd concentration remained unchanged regardless of the peat application; Figure 2b and Supplemental Figure S1). Similar observations on the concentration and accumulation of Cd in silage maize shoots were obtained in the CIS systems (Table 5). Generally, our results are in good agreement with the findings of Lin et al. [41] and Ma et al. [42], who also observed positive density-dependent uptake of Cd by Gallant soldier plants.

With or without peat amendment, with an increase in plant density, the RCI_inter_ value of Indian mustard increased by 8–37%, but that of silage maize decreased by 97–156% in the CIS systems (Supplemental Figure S3a). Hence, the facilitative effect of inter-specific interaction on silage maize exceeded its competitive effect on Indian mustard, resulting in increased silage maize shoot Cd concentration and accumulation with increasing density of Indian mustard (Table 5). However, when silage maize was mono-cropped (i.e., without inter-specific competition), Cd concentration and accumulation in silage maize shoots ranked the highest in the MS systems. In addition, there was an inverse relationship between the shoot Cd concentrations of Indian mustard and silage maize plants in a given plant density group (Figure 2b, Table 5). Therefore, it may be feasible to minimize Cd uptake by silage maize in the co-cropping systems. It also implies that silage maize should be co-cropped with Indian mustard at an appropriate plant density in Cd-polluted soils, in order to achieve a simultaneous remediation of Cd-contaminated soils and production of crops.

### 4.4. Effects of Peat Application on Plant Growth and Cd Uptake

Peat application can improve the physicochemical properties of soil, thereby affecting plant growth and Cd uptake. In the previous studies, soil amendment with peat showed varying effects [14], no effect [25] or positive effect on plant growth [13,26], depending on the peat application rate, soil type and plant species. In the present study, peat application significantly (*p* < 0.05 or <0.01) increased shoot biomass of Indian mustard (except in MI_4_WP vs. MI_4_NP; Figure 2a) and silage maize (Table 5).

A recent study has shown that the peat amendment of Pb/Zn tailings caused a substantial increase in the growth of annual herbaceous plants due to the enhanced N and P supply [43]. However, the effect of increased nutrient supply on biomass partitioning was shown to differ among plant species [33]. In the present study, it is noteworthy that the growth-promoting effect of peat application was more obvious in Indian mustard than silage maize (Figure 2a, Table 5). Furthermore, the R/S ratios of both species showed decreasing trends with the application of peat (Table S2). This result is consistent with the finding of Mašková and Herben [33], who observed that R/S ratio was lower in treatments with higher nutrient supply. Thus, it is suggested that with increasing nutrient supply, R/S ratio may be plastically altered in an adaptive way, whereby relatively more biomass can be allocated to shoots than to roots of both species grown in the peat-amended soils.

Although in mono-cropping of low-density Indian mustard or silage maize, the DTPA-extractable Cd concentration in soil significantly (*p* < 0.05) increased with the application of peat (Figure 1b), the Cd concentration in Indian mustard shoots significantly (*p* < 0.05) decreased in all peat-amended treatments, while that in silage maize shoots did not change significantly regardless of the peat application (Figure 2b, Table 5). This is largely because of the biodilution effect, which meant that the shoot Cd uptake of both plant species increased at a much lower rate in comparison with the increase in their shoot biomass, especially for Indian mustard plants. It seemed that soil amendment with peat did not influence Cd uptake by Indian mustard (Supplemental Figure S1), but promoted Cd accumulation by silage maize regardless of the density of Indian mustard (Table 5). Our results contradict the finding reported by He and Singh [25], but are in good agreement with the results of Shen et al. [13].

Interestingly, in the MS and CIS systems, both the shoot Cd removal rate and TF_Cd_ value significantly (*p* < 0.05) increased in the peat-amended treatments, as compared with those in the respective non-amended treatments (except in CI_4_S_1_WP vs. CI_4_S_1_NP, the TF_Cd_ value did not change significantly after applying peat; Figure 3b,d). At the very least, this indicates that whether silage maize was mono-cropped or co-cropped, soil amendment with peat significantly enhanced the shoot Cd removal efficiency from soil, but also greatly promoted the capacity to transport Cd from roots to shoots. In the previous study, Shen et al. [13] observed that the Cd removal rates by both shoots and roots increased following peat application, which is in partial agreement with our results (i.e., enhanced shoot Cd removal rates in most of the peat-amended treatments). As noted by the authors [13], the enhanced Cd availability in the peat-amended treatments can facilitate the transport of Cd due to the formation of soluble DOM-Cd complexes, resulting in a relatively high Cd removal rate. Moreover, in our study, lower root but higher shoot Cd removal rates were observed in most of the peat-amended treatments (Figure 3b, Table S2), indicating higher Cd translocation from roots to shoots after applying peat, which was more pronounced in the MS and CIS than MI systems (Figure 3d). Therefore, an advantage of using such a co-cropping system with peat amendment is to maximize Cd removal from soil by harvesting shoot tissues.

## 5. Conclusions

When Indian mustard and silage maize were co-cropped, the shoot growth of Indian mustard per pot was reduced considerably, as compared with that obtained in the mono-cropping systems. In contrast, the shoot biomass of silage maize plants was not affected by the presence of Indian mustard, regardless of the plant density. Due to its relatively higher R/S ratio, silage maize exhibited a greater capacity to absorb water and nutrients from the rhizosphere soil than Indian mustard, thereby suffering less from the inter-specific competition.

The Cd accumulation in both Indian mustard and silage maize shoots decreased significantly in the co-cropping in comparison with mono-cropping systems. Plant density played an important role in determining Cd uptake by silage maize. Along with an increase in the density of Indian mustard from 0 to 4 plants per pot, Cd uptake of silage maize decreased in the low-density and increased in the high-density groups. Therefore, co-cropping of low-density Indian mustard with silage maize is conducive to the simultaneous remediation of Cd-contaminated soils and production of crops.

Whether silage maize was mono-cropped or co-cropped, both the shoot Cd removal efficiency from soil and the translocation capacity of Cd from roots to shoots were greatly enhanced by the application of peat. This finding likely resulted (at least in part) from the interaction between organic matter and available Cd in the peat-amended soils. There is a need for in-depth studies to improve our understanding of the effect of organic matter on the phytoavailability of Cd in peat-amended soil.

## Figures and Tables

**Table 1:** Basic physicochemical properties of the paddy soil and woody peat used in the present study.

Property		Measured Value		Analytical Method
		Soil	Peat	
PSD (%)	Sand (2–0.05 mm)	18.6	—	Pipette method
	Silt (0.05–0.002 mm)	46.3	—	
	Clay (<0.002 mm)	35.2	—	
Texture		Silty clay loam	—	USDA soil texture triangle
pH_water_		5.07	5.21	Potentiometry
OM (g kg^−1^)		24.4	462	K_2_Cr_2_O_7_-H_2_SO_4_ oxidation method
CEC (cmol kg^−1^)		9.82	83.0	Ammonium acetate method
TN (g kg^−1^)		1.51	2.64	Kjeldahl method
TP (g kg^−1^)		1.32	0.136	Acid digestion and Mo-Sb colorimetry
TK (g kg^−1^)		14.0	15.0	Sodium hydroxide fusion method
Cd_T_ (mg kg^−1^)		1.72	BDL ^a^	Acid digestion and atomic-absorption spectrophotometry
Cd_DTPA_ (mg kg^−1^)		0.832	BDL ^a^	DTPA extraction and inductively coupled plasma atomic emission spectrometry

Abbreviations: PSD, particle size distribution; OM, organic matter; CEC, cation exchange capacity; TN, total nitrogen; TP, total phosphorus; TK, total potassium; Cd_T_, total Cd; Cd_DTPA_, DTPA-extractable Cd. ^a^ BDL indicates that the concentrations of total Cd and DTPA-extractable Cd in peat were below detection limits (0.01 mg·kg^−1^ for total Cd and 0.03 mg·kg^−1^ for DTPA-extractable Cd).

**Table 2:** The notations of treatments and their details.

Treatment Notation	Treatment Number	Planting Pattern			Peat Application	
		Mono- or Co-Cropping	Indian Mustard Density (Plant pot^−1^)	Group	Rate (g kg^−1^)	Group
CtrlNP	C_1_	no plants		Control	0	NP
MI_2_NP	T_1_	mono-cropping of Indian mustard (MI)	2 (low density)	MI-2	0	NP
MI_4_NP	T_2_		4 (high density)	MI-4	0	NP
MS_1_NP	T_3_	mono-cropping of one silage maize plant		MS	0	NP
CI_2_S_1_NP	T_4_	co-cropping of Indian mustard with one silage maize plant (CIS)	2 (low density)	CIS-2	0	NP
CI_4_S_1_NP	T_5_		4 (high density)	CIS-4	0	NP
CtrlWP	C_2_	no plants		Control	30	WP
MI_2_WP	T_6_	mono-cropping of Indian mustard (MI)	2 (low density)	MI-2	30	WP
MI_4_WP	T_7_		4 (high density)	MI-4	30	WP
MS_1_WP	T_8_	mono-cropping of one silage maize plant		MS	30	WP
CI_2_S_1_WP	T_9_	co-cropping of Indian mustard with one silage maize plant (CIS)	2 (low density)	CIS-2	30	WP
CI_4_S_1_WP	T_10_		4 (high density)	CIS-4	30	WP

**Table 3:** ANOVA (*p* values) of pH and available Cd (DTPA-extractable) in soil in different treatments.

Item	Planting Pattern	Peat Application	Planting Pattern × Peat Application
pH	<0.001	<0.001	0.647
Available Cd	0.655	<0.001	0.020

**Table 4:** ANOVA (*p* values) of dry biomass, Cd concentration, and Cd accumulation in different parts (shoots and roots), and root/shoot (R/S) ratio, Cd removal rate, and Cd translocation factor (TF_Cd_) of Indian mustard and/or silage maize plants in different treatments.

Item		Planting Pattern	Peat Application	Planting Pattern × Peat Application
Dry biomass	Indian mustard shoots	<0.001	<0.001	0.013
	Silage maize shoots	0.054	<0.001	0.397
	Total roots	<0.001	0.045	0.452
Cd concentration	Indian mustard shoots	<0.001	<0.001	0.005
	Silage maize shoots	<0.001	0.004	0.944
	Total roots	<0.001	<0.001	0.004
Cd accumulation	Indian mustard shoots	<0.001	0.057	0.064
	Silage maize shoots	<0.001	<0.001	0.080
	Total roots	<0.001	0.135	0.761
R/S ratio		<0.001	0.011	0.396
Cd removal rate	Shoots	<0.001	<0.001	<0.001
	Roots	<0.001	0.135	0.760
	Whole plants	<0.001	<0.001	0.002
TF_Cd_		<0.001	<0.001	0.003

**Table 5:** Differences in dry biomass, Cd concentration, and Cd accumulation in maize shoots in different treatments.

Treatment Notation	Planting Pattern	Peat Application	Dry Biomass (g pot^−1^)		Cd Concentration (mg kg^−1^)		Cd Accumulation (μg pot^−1^)	
MS_1_NP	MS	NP	18.0 ± 0.3	a	6.76 ± 0.72	a	122 ± 15	a
MS_1_WP		WP	21.3 ± 2.2		7.45 ± 0.42		172 ± 5	
CI_2_S_1_NP	CIS-2	NP	16.1 ± 1.9	a	4.59 ± 0.02	c	73.8 ± 8.8	c
CI_2_S_1_WP		WP	20.1 ± 1.2		5.18 ± 0.11		104 ± 6	
CI_4_S_1_NP	CIS-4	NP	19.1 ± 0.2	a	5.50 ± 0.16	b	105 ± 3	b
CI_4_S_1_WP		WP	21.1 ± 0.3		6.24 ± 0.46		131 ± 9	
*t*-test for the main effect of Peat								
*p* value			**		NS		*	

For explanation of treatment notations, see Table 2; data are expressed as means ± SD (*n* = 3); per column, different lowercase letters (e.g., a, b, c, etc.) indicate significant (*p* < 0.05) differences among the planting patterns based on the Tukey test; the asterisks indicate significant (* *p* < 0.05) or highly significant (** *p* < 0.01) differences, and NS indicates a non-significant (α = 0.05) difference between the NP and WP treatment groups.

## Data Availability

All supporting data have been included in this study, and are available from the corresponding authors upon request.

## Supplementary Materials

The following are available online at https://www.mdpi.com/article/10.3390/toxics9050091/s1, Table S1: Differences in dry biomass and Cd accumulation in the roots of Indian mustard and/or maize plants in different treatments. Table S2: Differences in root/shoot (R/S) ratio and root Cd removal rate of Indian mustard and/or maize plants in different treatments. Figure S1: Effects of planting pattern and peat application on Cd accumulation in Indian mustard shoots. For explanation of treatment notations, see Table 2. Data are expressed as means ± SD (*n* = 3); different lowercase letters indicate significant (*p* < 0.05) differences among the planting patterns (the interaction between planting pattern and peat application was non-significant) based on the Tukey test; NS indicates a non-significant difference (at α = 0.05) between the NP and WP treatment groups. Figure S2: Effects of planting pattern and peat application on shoot biomass per Indian mustard plant. For explanation of treatment notations, see Table 2. Data are expressed as means ± SD (*n* = 3); different lowercase letters indicate significant (*p* < 0.05) differences among the treatments based on the Tukey test. Figure S3: Relative intensity of inter-specific competition (RCI_inter_) between different species in response to co-cropping of Indian mustard with maize (a), and influence of increasing plant density on relative intensity of intra-specific competition (RCI_intra_) between plants of Indian mustard in the mono-cropping systems (b). For explanation of treatment notations, see Table 2. Data are expressed as means ± SD (*n* = 3); different lowercase letters indicate significant (*p* < 0.05 or < 0.01) differences between the low-density and high-density groups (a) as well as between the NP and WP treatment groups (b) based on the *t*-test.
