# Supplementary material for: Co-Cropping Indian Mustard and Silage Maize for Phytoremediation of a Cadmium-Contaminated Acid Paddy Soil Amended with Peat
Source: Toxics. 2021 Apr 21;9(5):91. doi: 10.3390/toxics9050091 (PMC8143100; doi:10.3390/toxics9050091)
Supplement: Supplementary file 1 [file toxics-09-00091-s001.zip › toxics-1177893-supplementary.pdf]

toxics

MDPI

Article

Supplementary Material: Co-Cropping Indian Mustard and Silage Maize for Phytoremediation of a Cadmium-Contaminated Acid Paddy Soil Amended with Peat

Sifan Wang, Yong Liu, Khalil Kariman, Jialin Li, Huihua Zhang, Fangbai Li, Yinglong Chen, Chongjian Ma, Chuanping Liu, Yuzhen Yuan, Zhiqiang Zhu and Zed Rengel

Table S1. Differences in dry biomass and Cd accumulation in the roots of Indian mustard and/or maize plants in different treatments.

Group Abbreviation	Planting Pattern	Peat Application	Dry Biomass ($g\ pot^{-1}$)		Cd Accumulation ($\mu g\ pot^{-1}$)	
MI$_2$NP	MI-2	NP	0.33 ± 0.03	d	4.61 ± 0.63	d
MI$_2$WP		WP	0.51 ± 0.06		2.81 ± 0.81	
MI$_4$NP	MI-4	NP	0.88 ± 0.12	c	16.6 ± 1.9	c
MI$_4$WP		WP	0.96 ± 0.30		13.5 ± 6.1	
MM$_1$NP	MM	NP	2.60 ± 0.12	b	42.7 ± 4.4	b
MM$_1$WP		WP	2.63 ± 0.12		41.8 ± 4.3	
CI$_2$M$_1$NP	CIM-2	NP	2.57 ± 0.22	b	41.4 ± 1.6	b
CI$_2$M$_1$WP		WP	2.77 ± 0.10		35.6 ± 2.1	
CI$_4$M$_1$NP	CIM-4	NP	3.30 ± 0.23	a	52.3 ± 3.9	a
CI$_4$M$_1$WP		WP	3.91 ± 0.75		52.4 ± 7.7	
t-test for the main effect of Peat						
P value			NS		NS	

For explanation of treatment notations, see Table 2; data are expressed as means ± SD (n = 3); per column, different lowercase letters indicate significant ($P < 0.05$) differences among the planting patterns based on the Tukey test; NS indicates a non-significant difference (at $\alpha = 0.05$) between the NP and WP treatment groups.

Table S2. Differences in root/shoot (R/S) ratio and root Cd removal rate of Indian mustard and/or maize plants in different treatments.

Group Abbreviation	Planting Pattern	Peat Application	R/S Ratio		Root Cd Removal Rate (%)	
MI$_2$NP	MI-2	NP	0.07 ± 0.00	b	0.04 ± 0.01	d
MI$_2$WP		WP	0.06 ± 0.00		0.03 ± 0.01	
MI$_4$NP	MI-4	NP	0.09 ± 0.01	b	0.16 ± 0.02	c
MI$_4$WP		WP	0.08 ± 0.01		0.13 ± 0.06	
MM$_1$NP	MM	NP	0.14 ± 0.01	a	0.41 ± 0.04	b
MM$_1$WP		WP	0.12 ± 0.01		0.41 ± 0.04	
CI$_2$M$_1$NP	CIM-2	NP	0.15 ± 0.03	a	0.40 ± 0.02	b
CI$_2$M$_1$WP		WP	0.12 ± 0.01		0.35 ± 0.02	
CI$_4$M$_1$NP	CIM-4	NP	0.15 ± 0.01	a	0.51 ± 0.04	a
CI$_4$M$_1$WP		WP	0.15 ± 0.03		0.51 ± 0.08	
t-test for the main effect of Peat						
P value			NS		NS	

For explanation of treatment notations, see Table 2; data are expressed as means ± SD (n = 3); per column, different lowercase letters indicate significant ($P < 0.05$) differences among the planting patterns based on the Tukey test; NS indicates a non-significant difference (at $\alpha = 0.05$) between the NP and WP treatment groups.

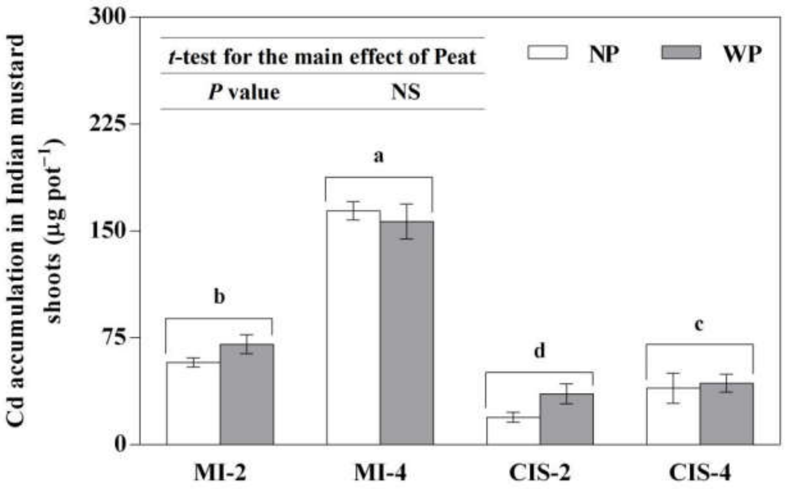

Figure S1. Effects of planting pattern and peat application on Cd accumulation in Indian mustard shoots. For explanation of treatment notations, see Table 2. Data are expressed as means ± SD (n = 3); different lowercase letters indicate significant ($P < 0.05$) differences among the planting patterns (the interaction between planting pattern and peat application was non-significant) based on the Tukey test; NS indicates a non-significant difference (at $\alpha = 0.05$) between the NP and WP treatment groups.

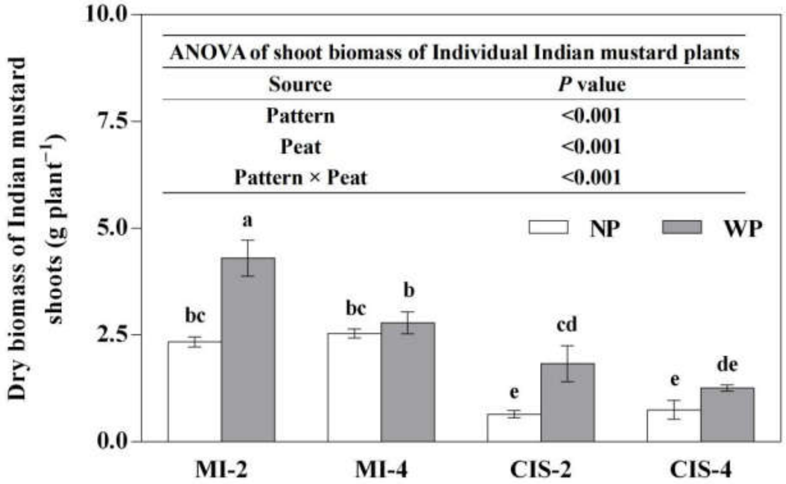

Figure S2. Effects of planting pattern and peat application on shoot biomass per Indian mustard plant. For explanation of treatment notations, see Table 2. Data are expressed as means ± SD (n = 3); different lowercase letters indicate significant ($P < 0.05$) differences among the treatments based on the Tukey test.

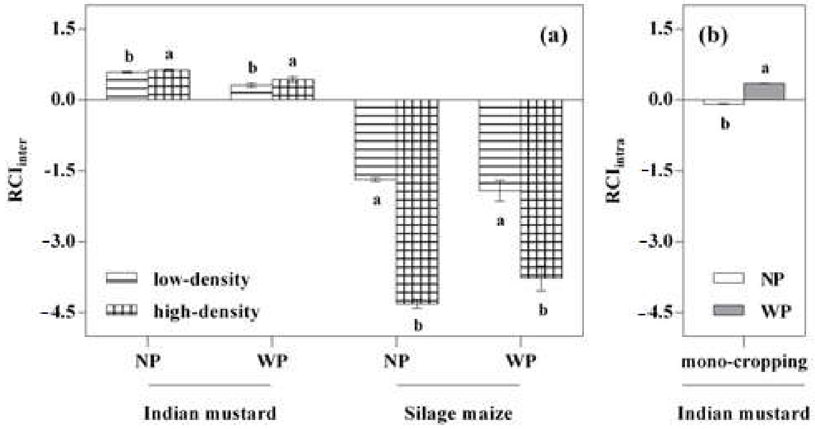

Figure S3. Relative intensity of inter-specific competition (RCI_{inter}) between different species in response to co-cropping of Indian mustard with maize (**a**), and influence of increasing plant density on relative intensity of intra-specific competition (RCI_{intra}) between plants of Indian mustard in the mono-cropping systems (**b**). For explanation of treatment notations, see Table 2. Data are expressed as means ± SD (n = 3); different lowercase letters indicate significant ($P < 0.05$ or < 0.01) differences between the low-density and high-density groups (a) as well as between the NP and WP treatment groups (b) based on the t-test.
